# Acoustic Emission Detection of Macro-Cracks on Engraving Tool Steel Inserts during the Injection Molding Cycle Using PZT Sensors

**DOI:** 10.3390/s130506365

**Published:** 2013-05-14

**Authors:** Rajko Svečko, Dragan Kusić, Tomaž Kek, Andrej Sarjaš, Aleš Hančič, Janez Grum

**Affiliations:** 1 Faculty of Electrical Engineering and Computer Science, University of Maribor, Smetanova 17, Maribor 2000, Slovenia; E-Mails: rajko.svecko@uni-mb.si (R.S.); andrej.sarjas@uni-mb.si (A.S.); 2 TECOS Slovenian Tool and Die Development Centre, Kidričeva 25, Celje 3000, Slovenia; E-Mails: dragan.kusic@tecos.si (D.K.); ales.hancic@tecos.si (A.H.); 3 Faculty of Mechanical Engineering, University of Ljubljana, Aškerčeva 6, Ljubljana 1000, Slovenia; E-Mail: tomaz.kek@fs.uni-lj.si

**Keywords:** injection molding, process monitoring, acoustic emission, PZT sensors, piezoelectric effect

## Abstract

This paper presents an improved monitoring system for the failure detection of engraving tool steel inserts during the injection molding cycle. This system uses acoustic emission PZT sensors mounted through acoustic waveguides on the engraving insert. We were thus able to clearly distinguish the defect through measured AE signals. Two engraving tool steel inserts were tested during the production of standard test specimens, each under the same processing conditions. By closely comparing the captured AE signals on both engraving inserts during the filling and packing stages, we were able to detect the presence of macro-cracks on one engraving insert. Gabor wavelet analysis was used for closer examination of the captured AE signals' peak amplitudes during the filling and packing stages. The obtained results revealed that such a system could be used successfully as an improved tool for monitoring the integrity of an injection molding process.

## 1. Introduction

**Figure 1.**
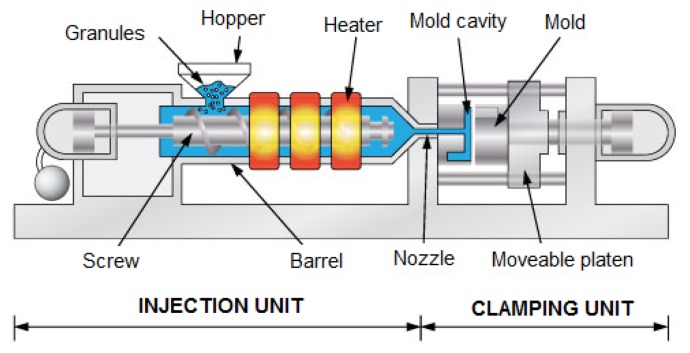
Simple schematic of injection molding machine.

Injection molding is one of the more commonly used processes in today's plastic manufacturing industry. The reason for this lies in its simple operation. Thermoplastic material, usually in the form of granules (or pellets), is fed into the injection molding machine through a hopper. At the entrance point of the granules into the cylinder, a screw rotates and moves them forward into the screw channels. The granules are then forced against the wall of a heated cylinder and then start to melt because of the conduction from the heating units mounted alongside the cylinder, and the friction-heat generated by the screw's rotation. The melt is transported to the screw's tip. When the desired volume of melt is obtained, the screw rotation stops. Then the injection stage and afterwards the packing stage start, where the clamp unit keeps the empty mold closed and the screw moves forward as a whole ram that forces the melt into the mold cavity. When the mold cavity is almost volumetrically filled, the screw is held in the forward position in order to maintain a holding pressure. At this time the melt cools down and volumetrically shrinks. This allows a little more melt to enter into the mold to compensate for the volumetric shrinkage of the thermoplastic material. During the cooling time the gate is completely frozen and the cavity pressure quickly drops to a very low value. The plastic product continues to cool down, and solidifies. After the cooling time has elapsed the plastic product becomes stiff enough for the mold to then be opened and the product ejected [1]. A simple schematic drawing of a typical injection molding machine is shown in Figure 1.

One of the main advantages of the injection molding process is the fact that it allows the high-volume manufacturing of plastic products. Often the geometric form of such products is very complex and must meet narrow tolerance requirements. This is especially true in those cases where different thermoplastic materials that have different shrinkage values are used.

In addition to the dimensional requirements, it is also very important that the product have no visible errors, such as voids, sink-marks, flash, *etc*. These errors usually occur due to an incorrect set of process parameters, such as injection pressure, holding pressure, injection speed, melt temperature, tool temperature, holding time, and cooling time.

The mold cavity pressure is usually measured when monitoring the qualities of the plastic products and the stability of the injection molding process during production. The mold cavity pressure is already widely recognized as a good quality indicator and therefore used by many researchers [2–4].

The first signs of macro-cracks on the surface of an engraving tool's steel insert can occur practically at some time during high-volume production. This leads to a visual error on the produced plastic product and is a type of mold defect. Under normal conditions it cannot be detected using cavity pressure measurements. One of the more popular and powerful methods for the real-time detection of macro-cracks is Acoustic Emission (AE), which is often described as a wave phenomenon. It is unique compared to all other nondestructive test (NDT) methods because it can detect and locate macro-cracks immediately as they occur.

In the past significant research work has been published in the field of detecting, monitoring, and diagnosing various defects during different manufacturing processes by using the acoustic emission method without any problems. Wilcox *et al.*, used the cutting force and acoustic emission signals for the monitoring of tool insert geometries during rough face milling. The tool insert geometries, produced by precision grinding, were intended for simulating different forms of naturally-occurring wear such as crater, notch and flank wear, local changes in rake angle, and edge breakdown. Their results indicated that both the cutting force and the AE signals can be used as indicators of changes in cutting tool geometry with consequent implications for diagnostic, geometry-specific, wear detection [5]. Li briefly presented various AE signal methodologies for tool wear monitoring during turning. He showed that careful processing of AE signals and their feature extraction perform an important step for improving and developing new tool wear monitoring systems [6]. Grum and Kek used the AE method for monitoring the laser-cut quality of steel plates with different thicknesses. They found that laser cutting conditions affected the measured AE signals due to interaction among laser light, a cutting gas, and a plate material, which produced different physical and chemical phenomena during the laser cutting process. Their experimental results confirmed that AE signals can be used successfully as quality indicators in laser cutting [7,8]. Gao *et al.*, applied AE testing for the fault diagnosis of low-speed heavy-duty gears. Through analysis of the measured AE data from the faults of on-spot low-speed heavy-duty gears they validated the redundant second generation wavelet transform during the processing and de-noising of AE signals. Authors have illustrated that the AE method can be used for the fault diagnosis of on-spot low-speed heavy-duty gears and that it can be a significant supplement to vibration testing diagnosis [9]. Li *et al.*, used three AE sensors for the monitoring and failure analysis of corroded bridge cables under fatigue loading. Their experimental results showed that the fatigue damage evolution process of the corroded bridge cable can be expressed according to the curves of the cumulative AE energy [10]. Posada-Roman *et al.*, developed and tested a fiber optic sensor for the acoustic detection of partial discharges in oil-paper insulated electrical systems. Their test results showed that the developed sensor had suitable sensitivity but still some further work had to be done on improving the stability of the optical fiber sensor head [11]. Alfaro and Cayo presented the relationship between welding quality and optical-acoustic emissions from electric arcs, during welding runs, in the GMAW-S process. Authors have developed data fusion algorithms by assessing known welding quality parameters from optical-acoustic emissions. The data fusion algorithms have shown positive results when detecting induced perturbations throughout the welding path despite the fact that acoustic monitoring was sensitive to environmental noise [12]. Li and Cao used the AE method for monitoring the damage of temperature fatigue load on polyvinyl alcohol (PVA) fiber concrete specimens. They found that temperature fatigue load had significant influence on PVA fiber concrete damage and AE characteristic parameters, such as the amplitude, energy, and time duration of an AE signal [13].

In the field of injection molding, some attempts have been made to build cheaper cavity pressure sensors [14,15]. The main idea was to replace the traditional quartz with PZT piezoceramic material but this failed in the end. The decisive factor was the higher stability of quartz sensors because there is no need for subsequent sensor recalibration.

This article investigated experimentally the possible presence of macro-cracks on two engraving inserts, using two resonant PZT sensors, by capturing AE signals during the injection molding of test specimens, which are used for the shrinkage evaluation of thermoplastic materials. The obtained AE results from both engraving inserts showed that this promising technique using PZT sensors can be successfully used for failure detection. Together with a cavity pressure sensor mounted inside the mold, they provide an improved measurement system for monitoring the injection molding process.

## 2. Experimental Setup

**Figure 2.**
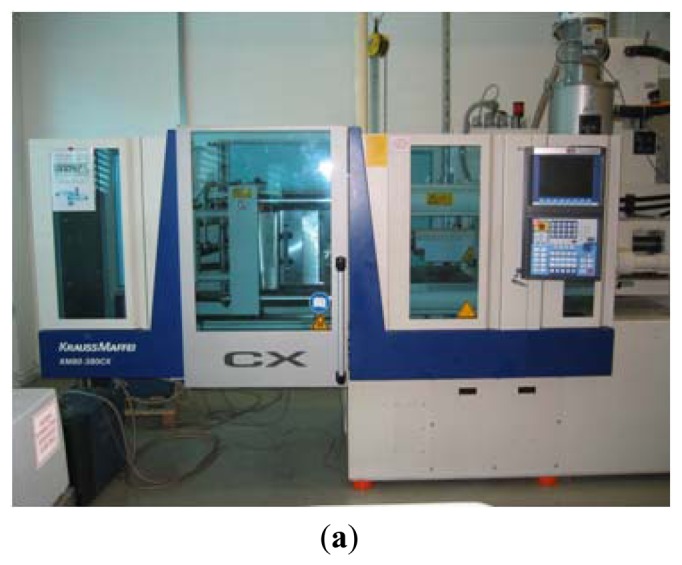

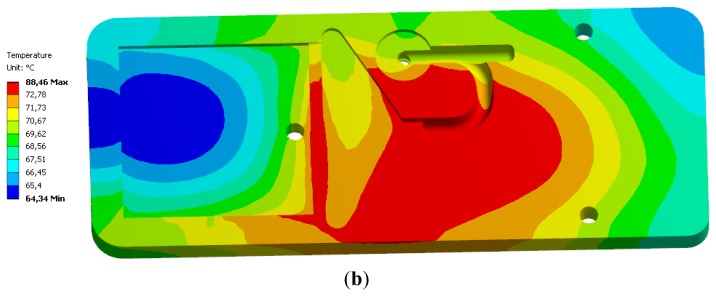
(**a**) A KraussMaffei injection molding machine; (**b**) Result of transient thermal analysis conducted on the engraving tool's steel insert.

All the practical experiments were carried out on a KraussMaffei type KM80SP380CX injection molding machine (Figure 2(a)), which had a 40 mm screw diameter and a maximal clamping force of 800 kN. A commercially-available low-cost polypropylene thermoplastic material (Isofil H40 C2 FNAT) was used during the experiments, which contains 40% by weight of calcium carbonate, and is widely used throughout the automotive industry. One cavity test mold defined by ISO 294-3 was used for the injection molding of standard D2 ISO test specimens with square dimensions of 60 mm × 60 mm and thickness of 2 mm [16]. Two engraving tool's steel inserts were used during the experiments, of which the first one was completely new and the second had already been used for some time. A proper selection of injection molding parameters was necessary before commencing the experimental tests. Therefore six injection molding parameters were chosen and appropriate values assigned to them, as listed in Table 1. We produced five test specimens with each engraving insert and evaluated the measured AE signals in each of the five cycles.

A transient thermal analysis on the engraving tool's steel insert was also conducted over one minute using two 230 W ceramic cartridge heaters, which were placed on the backside of the engraving insert. As can be seen in Figure 2(b), the temperature at the middle part of the engraving inserts can reach almost 90 °C and easily exceeds the defined set value of 60 °C because of the high thermal conductivity of the tool's steel. In our case this was very problematic and meant that we could not directly mount the acoustic emission PZT sensors onto the surface of the engraving tool's steel insert, since the maximal allowed temperature range is 100 °C.

**Figure 3.**
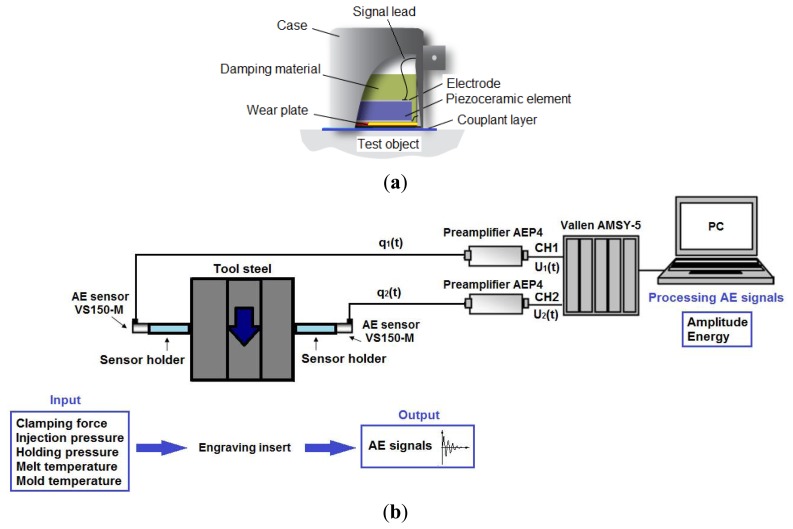
(**a**) Schematic diagram of a typical acoustic emission PZT sensor mounted on a test object; (**b**) Diagram of the experimental measurement of AE signals using two PZT sensors.

A typical structure of an acoustic emission PZT sensor is shown in Figure 3(a) [17]. The active element is always one type of the piezoelectric element that makes the transduction. A special ceramic in the form of a disc is mostly used for the fabrication of AE sensors, such as PZT (lead zirconate titanate), besides quartz and PVDF (polyvinylidene fluoride). This is acoustically coupled through electrodes, a wear plate, and a couplant layer, to the test object's surface from which the acoustic waves propagate to the piezoelectric element. One electrode is connected to the signal lead and the second to the electrical ground. A wear plate is used to protect the active element. The sensor case and damping material are used to minimize the electromagnetic interferences from the external environment.

In our experiments we used two VS150-M contact PZT sensors in order to capture acoustic emission signals. This sensor, which has a very high sensitivity, can detect acoustic emissions within a frequency range from 100 kHz to 450 kHz. The highest sensitivity is obtained at a resonant frequency of 150 kHz. Normally such sensors are used for testing the integrity of metal and composite structures. For example, Nair and Cai used four resonant AE sensors for monitoring bridge structures, while obtaining AE data from two field test cases under real load conditions. Three materials (steel, concrete, and fiber reinforced polymer) were considered during their research. They found that continued monitoring can help trace the health of a bridge construction because the intensity analysis technique assesses cumulative AE data over successive loads. The intensity charts additionally helped to better estimate the damage severity, although clearly marked zones of damage are not as yet prescribed for steel and concrete [18]. Kwon *et al.*, used a mesh of 14 resonant AE sensors to check the structural defects of a repaired storage tank. The AE signals were obtained and analyzed during a load sequence of 90%–110% maximum operating load range. They noticed that genuine emissions, which resulted from the weld defect areas, appeared as a band pattern in which the value of event counts was proportional to peak amplitude. The mechanical noise resulting from valve shut-off was characterized by low amplitude signals, a high event rate, and a high count. They concluded that the detected emissions from weld defect areas were active but not characteristic of causing structural defect growth [19]. Giordano *et al.*, developed a new method for the analysis of failure modes in polymer-composite materials by means of the AE method. Authors have used a single carbon fiber composite based on a polyester matrix as a sample model. A resonant AE sensor was used as a trigger for fiber breakage during the tensile loading test and the FFT analysis of signals, which was obtained from the wide band AE sensor, and was performed in order to evaluate their frequency spectrum content. Authors have shown that a clear frequency pattern could be characterized during different tensile tests [20].

In order to avoid this temperature problem we used, in the worst case when the temperature exceeded 90 °C, two sensor holders (acoustic waveguides) on which each of the two acoustic emissions PZT sensors were mounted because we had limited space for accessing the mounting-surface on the engraving insert. A good mechanical and thermal distance between the PZT sensor and the engraving insert was achieved using these sensor holders. A good acoustic coupling between the resonant sensors and the surfaces of the acoustic waveguides was accomplished using coupling silicon grease. The entire experimental setup is shown in Figure 3(b).

Both acoustic emission PZT sensors were connected through two AEP4 preamplifiers to the Vallen AMSY-5 measurement system, which was used to measure and export the captured AE signals. We recorded the AE signals on both engraving inserts in each cycle. According to the selected values for the injection molding parameters in Table 1, we set the appropriate AEP4 preamplifier gain to 40 dB. In this way we reduced the impact of noise on signal acquisition and avoided the need for analog or digital filter implementation. Wavelet analysis and all further processing of the captured AE signals were done in the Matlab programming language.

## 3. Background of Acoustic Emission Signal Processing

Acoustic emission testing uses the attributes of particular waves for characterizing a material or a structure in which waves are traveling. Amplitude, energy, and frequency are the usual waveform parameters that are regularly observed during acoustic emission tests.

In the case when a stress is applied to the injection mold engraving insert, an active flaw releases acoustic energy as an elastic waveform from the source (e.g., macro-cracks) throughout the structure. This can be further detected, located, and characterized by the AE measurement system. The acoustic emission signals are often analyzed using a Fourier transform (FT) or fast Fourier transform (FFT), in order to obtain frequency spectral information about the captured AE signals. A close examination of AE signals and their events are necessary because we want to know when a particular event took place. A problem occurs when using the classical Fourier transform, since we lose valuable information time regarding the particular event. In order to analyze a small section of the entire signal we can use the so-called short-time Fourier analysis (STFT), as presented by Gabor in 1946. The STFT windowing technique maps the signal into a 2-dimensional function of time and frequency. In this way the small sections of the entire signal are presented with time and frequency. The precision of such a technique is limited and can be determined by the size of the window. In order to improve the precision, it is necessary to vary the size of the window. An alternative for determining either the time or frequency content of the measured signal more precisely lies in the wavelet transform that needs to be preformed [21]. It has to be stated that Fourier transform is an excellent tool for analyzing the components of stationary signals. Furthermore, acoustic emission signals as such are non-stationary and transient signals. Therefore wavelet transform is a powerful technique for analyzing the components of such signals.

### 3.1. Gabor Wavelet Transform

By using wavelet transform, we can conduct a time-frequency transform of an acoustic emission signal, and separate the noise from the AE signal. Wavelet transform is, in fact, an extension of STFT. The basic functions of wavelet transform are not a constant window shape like in STFT but are scaled according to frequency. In general, a wavelet function *ψ(t)* is a complex-valued function. The theory and all details about wavelets can be found in Mallat's book [22].

The final results of the wavelet transform are wavelet coefficients, which are all functions of scale and position. A lot of time would need to be spent if we wanted to calculate the wavelet coefficients at every possible scale. A huge amount of coefficients is obtained at the end of calculations. In order to reduce the calculation time and the number of coefficients, choosing only a smaller number of scales and positions is recommended, based on the powers of two (dyadic scales and positions). This can easily be achieved by the usage of discrete wavelet transform. When a = 2^j^ and b = k 2^j^ (*j* and *k* are both integers), then the discrete wavelet in this case is defined as:

(1) $$\psi_{j,k}=2^{\frac{-j}{2}}\psi \;(2^{-j}t-k)$$

The discrete wavelet transformation of the sampled acoustic emission signal *S*(*n*) is obtained with:

(2) $$\text{DWL}\;(j,k)=\sum_{n\in Z}S\;\left(n\right)\cdot \psi_{j,k}$$

In our work we used a Gabor wavelet based on the Gaussian window function with a constant σ, as defined by:

(3) $$w\;(t-\tau )=\frac{\exp\left[-{\left(t-\tau \right)}^{2}\right]}{\sigma^{2}}$$

The mother wavelet with center frequency *ω_0_* and *” the constant Gabor shaping-factor $\gamma =\pi \cdot \sqrt{\frac{2}{\text{In}\;2}}=5.336$, is given by:

(4) $$\psi \;\left(t\right)=\frac{1}{\sqrt[{4}]{\pi }}\sqrt{\frac{\omega_{0}}{\gamma }}\exp\left[-\frac{t^{2}}{2}{\left(\frac{\omega_{0}}{\gamma }\right)}^{2}+i\omega_{0}t\right]$$

The selected constant value of 5.336 for the Gabor shaping-factor is commonly used in practice because it provides the best time-frequency resolution [23]. In this way we obtain fine time resolution at higher frequencies, whereas at lower frequencies appropriate frequency resolution is reached. In addition to this, Kurokawa et al., used the Gabor wavelet function to test the frequency filtering algorithms for source location on thin structures. They investigated the relationship between the Gabor shaping-factor *γ* by changing its value from 1.336 to 9.836, and the source location error. Their results confirmed that the minimal source location error (1.9 mm) was achieved with *γ* = *5.336* [24].

### 3.2. Crack Increase

In the cases of engraving tools' steel inserts, the flaws can appear in the forms of micro or macro-cracks, which cannot be avoided during the long-term production of plastic products when using different types of thermoplastic materials. We cannot be completely sure during daily production that a tool's steel material is flaw-free. Therefore we need to assume that a flaw of certain size will be present eventually. In order to evaluate the ability of an engraving tool's steel insert with a possible flaw when resisting a fracture, we need to firstly consider the flaw's size, the geometry of the insert, the loading conditions, and the used tool's steel material properties.

A stress-intensity factor KI is often used to determine the fracture toughness of the used tool's steel material. The numeral subscript I indicates mode one of the fracture, and is known as the condition where the crack-plane is normal towards the greatest tensile loading-direction.

Finally, on the basis of fracture mechanics, we can say that the crack length *l_c_* will increase according to the following equation:

(5) $$l_{c}=\frac{{K_{IC}}^{2}}{\pi G_{f}\;\sigma_{c}^{2}}$$

where *σ_c_* is the applied fracture stress, *G_f_* is the geometrical factor of the engraving insert, and *K_IC_* is the critical stress-intensity factor that a material can still withstand without fracturing. When the stress-intensity factor *K_I_* exceeds the critical stress-intensity factor *K_IC_*, then an unstable fracture occurs and the crack increases [25].

## 4. Experimental Results

**Figure 4.**
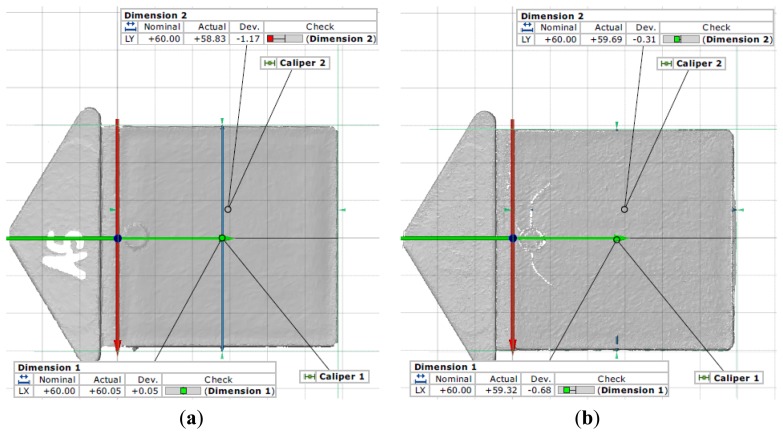
(**a**) Scan image of test specimen A5 with marked locations of width and length measurements; (**b**) Scan image of test specimen B5 with visible macro-crack and marked locations of width and length measurements.

Immediately after the production of both test specimens, we cut their runners in order to minimize any warpage. When both the test specimens had cooled down they were stored for 24 hours at a temperature of 23 °C. After the 24 hours, measurements on both test specimens were carried out at ambient temperature by using the ATOS II SO 3D scanning system which has an accuracy of a few hundredths of a mm. We measured the lengths and widths of both test specimens at selected locations, as shown in Figure 4. As can be seen in Figure 4(a), the nominal width was exceeded slightly by 0.05 mm, while all other measurements were within nominal values. The difference between the original dimensions (60 × 60 mm) and the measured dimensions after 24 hours indicated post-molding shrinkage in length and width, which could be calculated according to ISO 294-4 [26].

**Figure 5.**
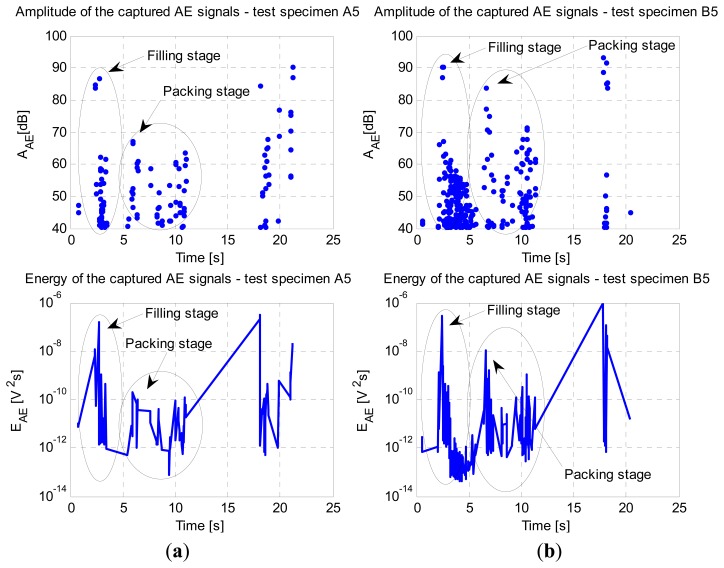
Intensities of AE events, amplitude, and energy of captured AE signals. (**a**) Test specimen A5 (new engraving insert); (**b**) Test specimen B5 (used engraving insert).

The acoustic emission signals were measured on-line during the injection molding cycle, as can be seen in Figure 5. A sampling frequency of 5 MHz was used. It can be noticed that the AE signals were largely generated during the filling and packing stages.

By comparing the intensities of the AE events on both test specimens, we can clearly see that there were more events generated during the production of test specimen B5. This result can normally be expected when using the AE method, which shows its great capability of detecting the macro-cracks on the engraving tool's steel insert. Additionally, we can notice in Figure 5(b), that the values for amplitude and energy during the filling and packing stages were significantly higher compared to those in Figure 5(a).

Acoustic emission signal intensity is proportional to signal energy [27], which is defined by an integral of the signal square, as shown in:

(6) $$E_{AE}=\int_{0}^{\infty }{\left|V(t)\right|}^{2}dt$$

Based on the captured AE signals in Figure 5, we focused closely on both test specimens' peak amplitudes during the filling and packing stages. In our case the captured AE signals are burst type. One of the more important features of the AE burst signal is the peak amplitude, which can be medium or high in the case of cracks. Therefore an appropriate short time-interval needs to be selected for a detailed analysis of AE signal burst.

**Figure 6.**
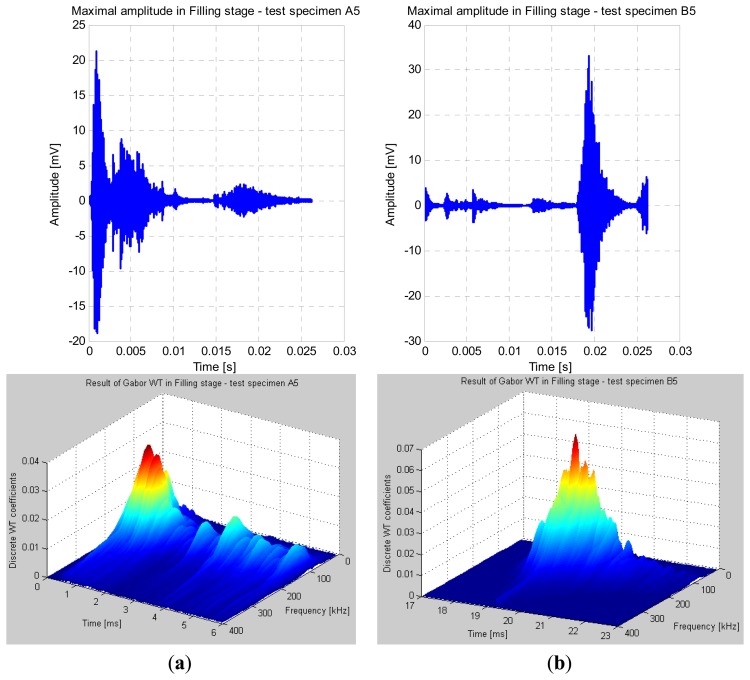
Maximal amplitudes and Gabor wavelet result during the filling stage. (**a**) Test specimen A5 (new engraving insert); (**b**) Test specimen B5 (used engraving insert with macro-crack).

In Figure 6(a,b), the discrete wavelet coefficients were calculated within a selected 6 ms time-interval just for the peak amplitude signals captured during the filling stage. In the case of test specimen A5, the maximal value of the calculated wavelet coefficients during the filling stage (0.033) was obtained at 150 kHz. At this frequency the sensitivity of the used acoustic emission PZT sensors was the highest. In the case of test specimen B5 at 100 kHz, the maximal value of the calculated wavelet coefficients during the filling stage (0.063) was obtained, and is a clear indicator for detecting defects on the engraving insert (e.g., presence of macro-cracks). The difference in values of the wavelet coefficients between the two test specimens during the filling stage was almost of a factor 2.

**Figure 7.**
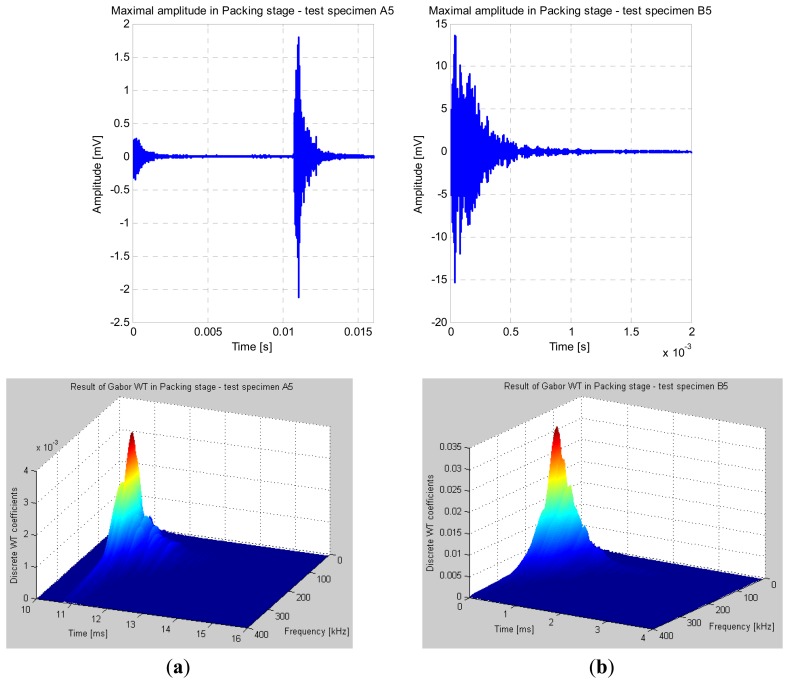
Maximal amplitudes and Gabor wavelet result during the packing stage. (**a**) Test specimen A5 (new engraving insert); (**b**) Test specimen B5 (used engraving insert with macro-crack).

The same procedure was used for evaluating the peak amplitudes during the packing stage. During the packing stage, an obvious difference could be noticed by comparing both amplitude values. In Figure 7(a) the wavelet coefficients were calculated over a 6 ms time-interval from 10 ms to 16 ms. For the test specimen A5, the maximal value of the calculated wavelet coefficients during the packing stage (0.003) was obtained at 120 kHz. In Figure 7(b) the wavelet coefficients were calculated over a shorter time-interval of 4 ms, which could be additionally reduced until reaching 1 ms in order to speed up the calculation and lower the total number of wavelet coefficients. For test specimen B5, the maximal value of the calculated wavelet coefficients during the packing stage (0.031) was obtained at 100 kHz. The difference in values of the wavelet coefficients was 10-fold between the two test specimens during the packing stage.

**Figure 8.**
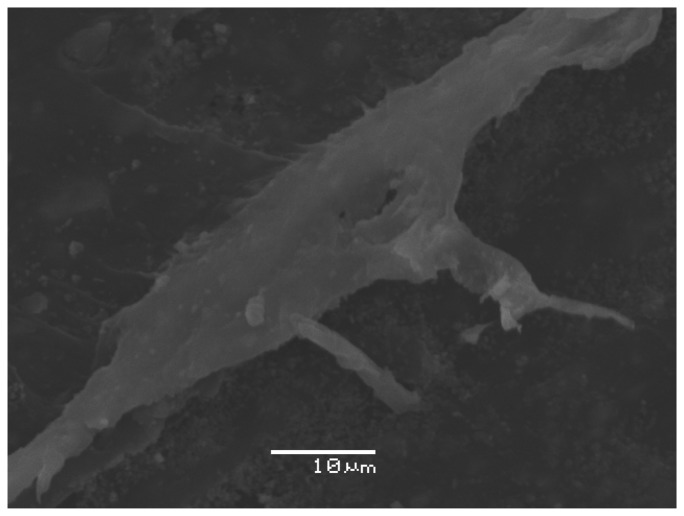
SEM image of the macro-crack on test specimen B5.

A surface morphological study of test specimen B5 was also done by a Scanning Electron Microscope (JEOL 5500 LV) using a secondary electron detector, and an accelerating voltage of 20 kV. Prior to commencing this study, the test specimen B5 was pre-cleaned with ethanol and dried for one hour at room temperature. Then the test specimen B5 was coated with a highly conductive film of gold by using a sputter-coater BAL-TEC SCD 500. The obtained scanning electron microscopy image at 2000× magnifications from the macro-crack on test specimen B5 is shown in Figure 8. As can be seen the width of the macro-crack is within the range from 5 μm to 20 μm. The total length of the macro-crack on test specimen B5 is around 30 mm. At the macro-crack location, the calcium carbonate filler is clearly visible and can be distinguished from the rest of the polypropylene material.

## 5. Conclusions

This paper experimentally investigated the presence of macro-cracks on two engraving inserts during the injection molding cycle using contact acoustic emission PZT sensors. We were able to detect from the captured acoustic emission signals on both engraving tools' steel inserts under the same processing conditions, the presence of a macro-crack on one of the engraving tools' steel inserts. On the defective engraving tool's steel insert we noticed a higher number of AE events, higher amplitude and energy values of AE signal during the filling and packing stages compared to those from the undamaged engraving tool steel insert. We used Gabor wavelet analysis to conduct a local analysis of the captured AE signal, which was a large signal that presented an entire production cycle of almost 22 seconds. By observing in depth over a small time-scale, the peak AE signals during the filling and packing stages, we found within both the time and frequency domains that the calculated discrete wavelet coefficients can additionally serve as quality indicators for engraving tool steel inserts. In the case of defective engraving tool steel insert, the values of the discrete wavelet coefficients were clearly higher, *i.e.*, almost double during the filling stage and 10-fold during the packing stage, compared to the non-defected engraving insert. By scanning both test specimens' surfaces we located the macro-crack on test specimen B5. The SEM observation of his surface confirmed that the macro-crack was the main reason for the higher number of AE events, amplitudes, and energy values of the captured AE signal during its production. According to our results we can declare that contact PZT sensors for measuring acoustic emission can be successfully used for monitoring the integrities of different manufacturing processes in real-time. This is one of the main advantages of the acoustic emission method in which PZT material plays an important role compared to all other NDT techniques.

## Figures and Tables

**Table 1.** Selected injection molding parameters with corresponding values.

**Injection Molding Parameters**	**Value**
Melt temperature	230 °C
Injection speed	45 mm/s
Injection pressure	1,100 bar
Packing pressure	400 bar
Packing time	5 s
Cooling time	10 s
